# Can natural killer cells cure leukemia?

**DOI:** 10.1038/s41375-026-02992-5

**Published:** 2026-06-15

**Authors:** Frank Cichocki, Martin Felices, Anders W. Matson, Madeline J. Lee, Jeffrey S. Miller

**Affiliations:** Department of Medicine, Masonic Cancer Center, University of Minnesota, Minneapolis, MN USA

**Keywords:** Leukaemia, Immunotherapy

**Fig. 1:**
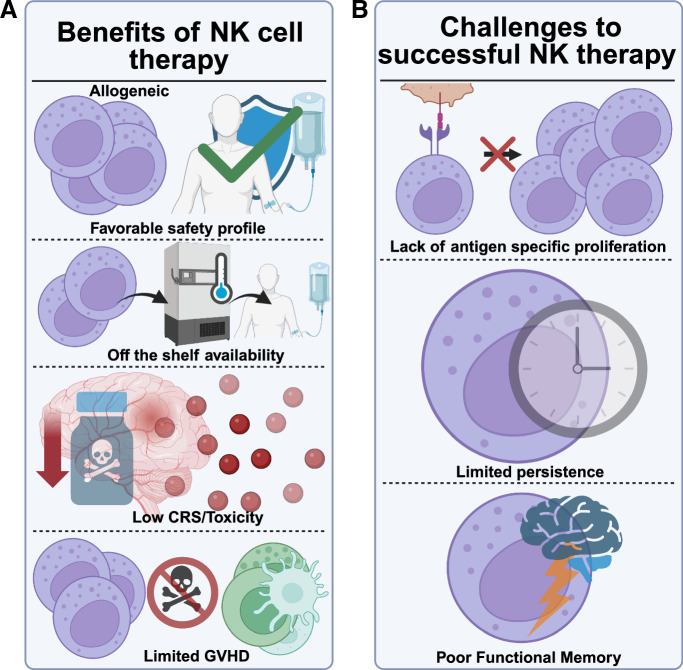
Comparisons of the benefits and challenges of NK cell versus T cell therapies. **A** Key advantages of NK cell-based therapies include increased safety profiles, reduced risk of GVHD, and the ability to manufacture allogeneic or universal donors for off-the-shelf use. **B** Major limitations of current NK cell therapies center on their restricted antigen-specific proliferation, limited in vivo persistence, and incomplete memory-like responses against AML.

As of 2025, 140 companies, including some in big pharma, were developing 160 NK cell strategies, including NK cell products (mostly allogeneic), NK cell engagers, and cytokines or other proteins to better unleash the function of NK cells (https://www.delveinsight.com/infographics/nk-cell-therapy-pipeline-insight). While sound basic and clinical investigation has shown that NK cell infusions have robust anti-tumor activity and are undoubtedly safer than T cells without graft-versus-host disease (GVHD) or cytokine release syndrome (CRS), they have limitations that have not been fully overcome to sustain industry investment for commercialization (Fig. 1). The clinical promise is supported by many small academic institutional clinical trials and even some from industry [1], many of which are focused on treating hematologic malignancies. While not all published, we estimate that several thousand cancer patients have been treated with an NK cell-based strategy over the past 2 decades worldwide against a variety of cancers.

**Fig. 2:**
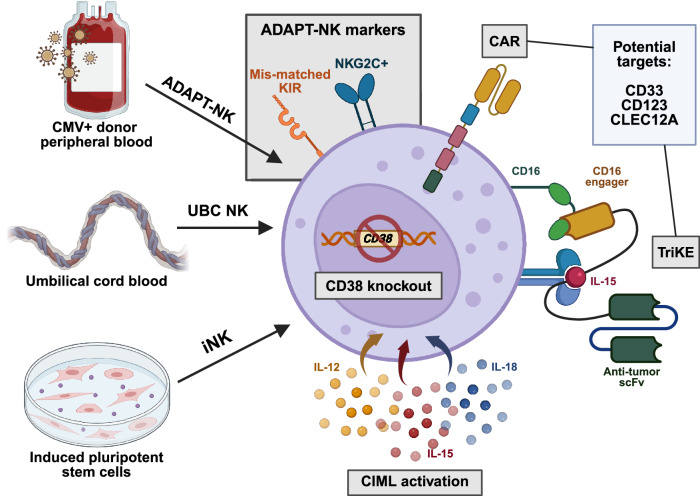
Schematic illustrating promising methods to overcome current limitations of NK cell therapeutics for leukemia. Left: 3 key sources for NK cells that can be used in adoptive cell therapies (peripheral blood from CMV+ donors, umbilical cord blood, and induced pluripotent stem cells). Center and right: 5 methods to enhance NK cell antitumor efficacy in leukemia. These are (1) the use of CMV+ donor “ADAPT-NKs”, which express high levels of NKG2C and are selected for patient-mismatched KIR expression; (2) chimeric antigen receptor (CAR) constructs targeting tumor antigens such as CD33, CD123, or CLEC12A; (3) tri-specific killer engager (TriKE) molecules that provide signaling to NK cells through CD16 and the IL-15 receptor complex while directing NK cell killing at tumor antigens; (4) cytokine-induced memory-like (CIML) activation of ex vivo NK cells via IL-12, IL-15, and IL-18; and (5) knockout of the *CD38* gene.

Our first non-gene edited single donor-sourced haploidentical NK cell trial was published 20 years ago, resulting in complete remission (CR) of up to 50% in patients with relapsed/refractory (r/r) acute myeloid leukemia (AML) [2–4]. These findings have been verified by other groups with NK cells expanded from umbilical cord blood [5], peripheral blood [6, 7], placenta (NCT02781467), and renewable induced pluripotent stem cells (Fig. 2) [1]. After our initial simple overnight activation with IL-2 or IL-15, additional strategies have been developed to generate cytokine-induced memory-like (CIML) NK cells to achieve a 44% CR rate in r/r AML [7]. A 58% CR rate in r/r AML was achieved using engineered feeders to expand NK cells to enable larger multi-dosed strategies [8]. When combining NK cells from the same donor used for allogenic transplantation, therefore, in a setting where the NK cells are autologous to the reconstituting immune system, NK cells can persist longer and avoid rapid allogeneic rejection [9]. While promising, clinical experience to date leads us to conclude that antigen-driven proliferation, long-term persistence, and functional memory will be needed for NK cell therapy to be durably efficacious for commercial approval (Fig. 1).

Learning from clinical trials of CD19-targeted chimeric antigen receptor (CAR) T cell (CTL019) in chronic lymphocytic leukemia (CLL) [10, 11], a highly informative retrospective study was performed after the completion of these trials to identify determinants of response and resistance. Responses were not related to patient age, peripheral tumor burden, prior therapy, or other common factors. Patients who achieved CRs exhibited dramatic in vivo expansion of CTL019 cells coincident with B cell aplasia in the first two weeks after infusion. In contrast, nonresponding (NR) patients displayed limited or, in most cases, no in vivo T cell proliferation and far less B cell aplasia. In extended follow-up analyses, CRs were observed beyond 5 years, and these patients still possessed detectable CTL019 cells in the peripheral blood with ongoing B cell aplasia. In addition to expansion and persistence, the frequencies of CD45RO^-^CD27^+^CD8^+^ T cells from CR patients at the time of leukapheresis were significantly higher than those observed in PR/NR patients [12]. The CD45RO^-^CD27^+^ subset of CD8^+^ T cells persists in a resting state and possesses properties of long-lived memory cells [13]. Therefore, long-lasting remissions were associated with an initial enrichment of long-lived memory cells, dramatic in vivo expansions, and functional persistence [12]. By analogy, we believe that these same principles are key to unlocking transformative advances in NK cell immunotherapy.

NK cells are close relatives of CD8^+^ T cells and mediate direct cytolysis of malignant cells through engagement of germline-encoded activating receptors [14]. As members of the innate immune system, most NK cells differ from CD8^+^ T cells in two crucial ways: they do not undergo rapid clonal expansion in response to activation, and they do not persist long-term. The absence of attributes definitional to adaptive immunity is a likely reason for the general lack of long-term remissions observed in patients receiving NK cell immunotherapies to treat r/r disease. For NK cells to rival their T cell counterparts in the immunotherapy space, they will need to exhibit antigen-driven expansion and persist long-term as a functional memory pool (Fig. 1). In other words, NK cells must be able to act like T cells.

Fortunately, human NK cells are remarkably diverse [15], and there is at least one context in which NK cell memory is formed. In both mice and humans, infection with cytomegalovirus (CMV) can result in robust clonal expansions of antigen-specific NK cells that maintain natural cytotoxicity and persist for months and likely years at stable frequencies [16–20]. NK cells exhibiting these hallmark features of lymphocyte memory are commonly referred to as “adaptive”. Intriguingly, adaptive NK cells and effector CD8^+^ T cells share overlapping whole genome epigenetic profiles [17], suggestive of a distinct developmental path for adaptive NK cells. Reactivation of CMV after reduced intensity hematopoietic cell transplantation in patients with advanced hematological malignancies is associated with significant expansions of adaptive NK cells and better disease-free survival, accounted for by relapse protection [18, 21].

Adoptive transfer of ex vivo expanded adaptive NK cells is one promising approach with immediate translational potential. CMV seropositive “superdonors” harbor large NKG2C^+^ adaptive NK cell subsets with single self-KIR expression. These NK cells, referred to as “ADAPT-NK cells”, can be expanded up to 1000-fold ex vivo with engineered feeder cells and exhibit efficient alloreactivity against tumor cell lines in vivo (Fig. 2) [22]. The process for generating ADAPT-NK cells has been scaled up under GMP conditions, and a clinical trial treating patients with r/r acute myeloid leukemia is planned at our institution and at the Karolinska Institute. Homozygous HLA-C1/C1 or HLA-C2/C2 patients will receive infusions of ADAPT-NK cells with single self-KIRs mismatched for HLA-C. Notably, CD16 and its co-stimulatory receptor CD2 remain highly expressed by ADAPT-NK cells, enabling future translational efforts to augment natural cytotoxicity with potent antibody-dependent cellular cytotoxicity responses using existing therapeutic monoclonal antibodies or Tri-Specific Killer Engager (TriKE) molecules developed by our group (Fig. 2) [23, 24]. These engagers agonistically bind to CD16 on NK cells and to CD33 or CLEC12A on AML blasts while delivering IL-15 to the immune synapse. This approach may allow endogenous NK cells to become antigen-specific in vivo or to be used in combination with an NK cell infusion product as described with a CD30 NK cell engager in combination with allogeneic NK cells in Hodgkin’s Disease [25].

Long-term efforts continue by our group to generate induced pluripotent stem cell (iPSC)-derived NK cells (termed “iNK”) with attributes of immunological memory. The iNK cell platform has several important advantages over traditional cellular immunotherapy products, including production of a renewable cell source, the ability to perform highly efficient multiplex genetic engineering, and the capacity to generate billions of cells from a single production run for off-the-shelf use (Fig. 2) [26]. Recently, we found that adaptive NK cells differ from their canonical counterparts with respect to expression of CD38, which is an ectoenzyme with nicotinamide adenine dinucleotide (NAD^+^) glycohydrolase activity [27]. Low-to-absent expression of CD38 on the surface of adaptive NK cells correlated with increased intracellular concentrations of NAD^+^ relative to canonical NK cells and resistance to oxidative stress-induced cell death. CRISPR-mediated deletion of *CD38* in iNK cells supported these findings, with *CD38* knockout iNK cells exhibiting elevated intracellular NAD^+^ levels, marked resistance to oxidative stress, and broad changes in gene expression mirroring the transcriptional profile of adaptive NK cells [28].

In our opinion, NK cell immunotherapy for the treatment of various types of cancers could match or potentially exceed results seen with CAR T cells, with a better safety profile and at a fraction of the cost. While the above studies represent steps in the right direction, a better understanding of the ontogeny of adaptive NK cells and the mechanistic bases for their ability to undergo antigen-induced clonal expansion and persistence long-term is needed to develop new NK cell-based immunotherapies that will achieve complete and durable remissions. Next-generation iNK cells with characteristics of immunological memory could be further engineered for the prevention of allogeneic rejection and for antigen-specific effector function with the incorporation of CARs. Our group is currently developing new CAR strategies optimized for high expression and function in NK cells, and newly developed CAR iNK cell products could be combined with TriKEs for enhanced potency. A Phase I dose finding study of our anti-CD16/IL-15/anti-CD33 TriKE for the treatment of adults with r/r AML is currently enrolling patients at the University of Minnesota (NCT06594445).

In summary, there have been remarkable advances in NK cell immunotherapy over the past decade, with many breakthroughs moving from the bench to the bedside, thanks to collaborative partnerships between industry and academia. Collectively, this work provides a foundation for continued advancement through the next decade and beyond. Significant challenges still lie ahead, but we know from the CAR T cell experience what the parameters are that define clinical success versus failure. The keys to success are antigen-driven proliferation, long-term persistence, and functional memory. We would be well served to keep this in mind as the NK cell immunotherapy field evolves into the future.
